# Family-supportive supervisor behaviors and career sustainability of e-commerce female workers: A mixed-method approach

**DOI:** 10.3389/fpsyg.2022.992458

**Published:** 2022-09-27

**Authors:** Huan Luo, Fa Li, George Kwame Agbanyo, Mark Awe Tachega, Tachia Chin

**Affiliations:** ^1^School of Business, Honghe University, Mengzi, China; ^2^College of Economics and Management, Taiyuan University of Technology, Taiyuan, China

**Keywords:** FSSB, career sustainability, self-efficacy, females, mixed-method approach

## Abstract

Women play an essential role in promoting societal and economic harmony development. However, compared with their male counterparts, female employees usually have to take on more family responsibilities while they endeavor to perform well at work. It is inevitable for them to face work–family conflicts; therefore, how to make female employees' careers more sustainable is a critical concern. Even though female career sustainability is well-explored in the literature, the combined effect of worker self-efficacy and family-supportive supervisor behaviors (FSSBs) on female career sustainability remains unexplored. To fill this gap, this study examines the influence of FSSB on female employee career sustainability, as well as the mediating role of self-efficacy. Moreover, a mixed-method approach was used to test the proposed relationships. The results indicate that FSSB has a positive impact on female career sustainability. Furthermore, the findings revealed that FSSB promotes female career sustainability through self-efficacy. This study establishes a theoretical framework for further research on the relationships between leader behavior and employee career sustainability. At the same time, it offers practical implications for supervisors in the management of subordinate career sustainability.

## Introduction

Female employees have become an indispensable workforce in the global labor market because they often display greater care, compassion, and circumspection at work than male workers (Hackett and Betz, 1981). Along with the fast growth of the e-commerce industry of China in recent years, various new types of e-commerce companies such as short video and live streaming have been booming, particularly amid COVID-19, which has brought unprecedented job opportunities. An increasing number of female workers have taken this golden opportunity to start their careers in relevant domains.

However, female workers face far greater challenges than male workers because of the following reasons. The first reason is interrupted career caused by physiological factors. Women's career psychology and working timeline experience constant interruptions by monthly cycles and pre-natal, natal, post-natal, and menopausal transitions. The unavoidable natural disposition of maternity, nursing, and parenting compilations definitely causes an effective impairment to the choices facing women, especially in terms of career sustainability (Grunert and Bodner, 2011). In addition, another challenge faced by women is work–family dual responsibilities (Betz and Hackett, 1997). Professional women have to take more responsibility for the family while trying to obtain certain work achievements. It is a challenge for women to meet the demands of multiple roles of work and family with limited time and energy. Last but not least, women face sociocultural gender belief challenges. Contrary to women's identity, competence, and experience for career preferences, traditional social perceptions of motherhood remain ubiquitous and, consequently, shape society's perspectives toward women's roles in the family (Bosch et al., 2018). A saying in the Chinese culture has it that “Man is in charge of breadwinning, and woman is in charge of homemaking”. Gendered conceptions about women's roles in the workforce, expectations for gendered jobs, and expectations regarding parenthood mean certain women obtain fewer promotion opportunities, lower salaries, and unequal employment terms (Bosch et al., 2018).

Women's ability to balance simultaneous obligations at home and work indeed determines their continuous long-term steady employment. The COVID-19 outbreak has triggered a global unemployment crisis, leading to concerns regarding career sustainability (Staniškiene and Stankevičiute, 2018). Individuals with sustainable professions have a constant source of income and are able to meet their demands, while disruptions of a job can lead to loss of livelihood and stress (Chin et al., 2019a). Many academics have undertaken the theoretical study of career sustainability from the standpoint of individuals (Richardson et al., 2017; Chin et al., 2019b), but few organizational factors have been involved (Chin et al., 2019a).

FSSB, as one of the special kinds of behavior of supportive leadership, puts more emphasis on the support (such as emotional supports and instrumental support) by supervisors to help subordinates alleviate the work–family conflict and balance work and family relationships (Thomas and Ganster, 1995; Hammer et al., 2009). Prior studies of FSSB are more related to its influences on individuals' work outcomes and health outcomes, such as higher job satisfaction (Odle-Dusseau et al., 2012; Bagger and Li, 2014), more work engagement (Qing and Zhou, 2017), more innovative work behaviors (Bamber et al., 2017), lower turnover intentions (Hill et al., 2016; Asghar et al., 2018; Zhang et al., 2020), less stress (Bouleh et al., 2022), and burnout–exhaustion (Komlenac et al., 2022). In addition, several studies regarding FSSB involve in the discussion of gender difference (Bosch et al., 2018; Jolly et al., 2021; Yu et al., 2022), and some of these researches also extend to various industries (Jolly et al., 2021; Sahin et al., 2021). However, few studies have provideda direct link between FSSB and career sustainability.

Landmark studies in the field of self-efficacy (Bandura, 1977; Hackett and Betz, 1981; Multon et al., 1991) utilize quantitative analysis to demonstrate that self-efficacy is an avoidable predictor of career goal achievement. Thus, a person's self-efficacy, to a large extent influenced by sociocultural beliefs, determines the person's degree of career satisfaction. Social constructivism (Palincsar, 1998), similar to the social cognitive theory and the self-efficacy theory (Bandura, 1977), demonstrates the mechanism of sociocultural interactions in influencing the establishment of one's self-efficacy with no regard to gender. However, while Hammer et al. (2009) conceive FSSB as an instrumental booster of self-efficacy, Huffman et al. (2017) found gender as an antecedent of subordinate perceptions, with family support being relatively advantageous for female employees.

To the best of our knowledge, even though elaborate research has been conducted on the interaction between FSSB, individual performance, and self-efficacy, few studies elucidated the direct link between FSSB and female workers' career sustainability, especially in the Chinese context. This study aimed to fill this gap by employing a mixed-method approach to investigate the impact of FSSB on female workers' career sustainability in the e-commerce industry in the Chinese context. In addition, self-efficacy is considered a mediator to impact the relationship between FSSB and career sustainability.

## Hypothesis development

### FSSB and female employee career sustainability

Defined as the behavior exhibited by supervisors who are supportive of families (Thomas and Ganster, 1995; Hammer et al., 2009), FSSB positively correlates with subordinates' perceptions of supervisor support for the family (Crain and Stevens, 2018). Regarding the construct of FSSB, Hammer et al. (2013) developed four specific dimensions: emotional support, instrumental support, role modeling, and creative work–family management. Both antecedents and outcomes of FSSB were identified by previous scholars, which indicated antecedents including supervisor individual-level factors and contextual-level factors and the outcomes of FSSB described from employee and team levels (Straub, 2012). Employee-level outcomes of FSSB include wellbeing (Kossek et al., 2018), job satisfaction (Bagger and Li, 2014), job performance (Mills et al., 2014), organizational commitment (Chambel et al., 2022), and turnover intentions (Asghar et al., 2018). Team-level outcomes include team performance (Hill et al., 2016) and team cohesion.

In the volatile career market, accentuated by the COVID-19 pandemic, career sustainability has become a growing issue for individuals, companies, and societies (Lawrence et al., 2015). Career sustainability is defined as sequences of career experiences reflected through a variety of patterns of continuity over time (De Vos et al., 2018). Newman (2011), Nagy et al. (2019), and Chin et al. (2022) proposed a conceptual model of career sustainability advising four groups of indicators of a sustainable career, namely, flexibility, renewability, integration, and resourcefulness, which can be applied as antecedent variables of a sustainable career.

Leader behavior plays a significant role in an employee's career, especially for female employees (Sargent et al., 2021). We assumed that FSSB has a significant impact on career sustainability because of the following reasons. First of all, working time and location restructuring and adjustment by creative work–family management could increase career satisfaction (Hwang and Ramadoss, 2017), decrease emotional exhaustion (Komlenac et al., 2022), and enhance career flexibility. Second, if family-supportive leaders provide emotional and instrumental support for female staff when work–family conflicts occur (Russo et al., 2015), women's capabilities of handling difficulties could be renewed in the process of resolving the conflicts, and they would reposition themselves; thus, career renewability is reinforced. Furthermore, role modeling from supervisors could help female workers learn experience, information, and knowledge from others (Epstein et al., 2015) and then integrate those pieces of knowledge to cope with the challenges in work and family, which would strengthen career integration. Finally, family-supportive supervisors are willing to provide resources for female workers to accomplish goals, which helps them keep enthusiasm for their job (Sahin et al., 2021) and involve themselves in the organization's activities unabatedly (Chambel et al., 2022). Taken together, the ongoing argument leads to the following hypothesis:

*Hypothesis 1: FSSB positively affects female employee career sustainability*.

### FSSB and female employee self-efficacy

As the core component of self-concept, self-efficacy refers to people's confidence or belief in their ability to achieve behavioral goals in a specific field (Schwarzer et al., 1997, Bandura, 1977). The accomplishment of a task can boost personal self-efficacy, whereas repeated failures can lower it. Also, observing a third person's achievement has an important influence on an individual's self-efficacy. Self-efficacy is also affected by verbal persuasion based on someone's direct or indirect experience. Furthermore, individuals' physical and mental reactions and emotions in the face of a job assignment will impact their performance, altering their sense of self-efficacy.

In reference to the previous section, family-supportive leadership has a positive influential impact on the formation of employees' self-efficacy (Mills et al., 2014). By adopting innovative management methods such as flexible working time, changing workplace, and an agile working approach, a family-supportive leader gives a boost of assistance to female employees, making them handle work and family affairs easier (Seong, 2016), and play the dual roles of work and family better; thus, female staff could gain the success experience, and self-efficacy becomes stronger. Furthermore, the self-efficacy of female employees also could be improved by observing the way a family-supportive supervisor overcomes difficulties at work. At the same time, persuasion and communication from that supervisor enhance the belief of female employees that they can deal with issues at work. At last, Mills et al. (2014) indicated that FSSB could bring positive health outcomes, such as the subjective wellbeing and confidence of subordinates being increased, while exhaustion (Koch and Binnewies, 2015), depression, and stress (Yragui et al., 2016) are reduced. When female employees with good physical and mental health can maintain a positive attitude, they will work with confidence and a good mood (Betz and Hackett, 1997), and their self-efficacy to complete tasks will be augmented accordingly (Grunert and Bodner, 2011). Thus, the following hypothesis is proposed:

*Hypothesis 2:FSSB positively affects female employee self-efficacy*.

### Self-efficacy and female employee career sustainability

The literature has explicitly documented the high correlation between cognitive/intellectual capital and employee motivation and achievement. As the perception of one's capability to fulfill a task successfully, self-efficacy is a principal element of career decision (Hackett and Betz, 1981). The perception of “I can do it” is a prerequisite factor for individuals to achieve a certain goal. Self-efficacy determines how much effort a person puts into a task and how long the person is able to persist. An individual with a strong sense of self-efficacy works harder to tackle challenges, while those with a low sense of self-efficacy may reduce their efforts or even drop out of their career objectives. The concept of career decision-making self-efficacy (Taylor and Betz, 1983), developed by prior research, was demonstrated as a critical factor for ensuring a sustainable career containing job satisfaction, intrinsic satisfaction (Peng and Mao, 2015), career choice commitment (Chen et al., 2021), and career exploration (Rogers et al., 2008). When female employees have a strong sense of self-efficacy toward a career, they maintain more interest and motivation in work and believe that they can effectively complete the job assignment and deal with difficulties in work through their own ability and knowledge; therefore, both job satisfaction and career commitment will be strengthened. Even if demission happened because of some unpredictable reason, those female employees would still have the competencies and confidence to start a new job and make their career sustainable. Therefore, we hypothesized the following.

*Hypothesis 3: Self-efficacy positively affects female employee career sustainability*.

### The mediating role of self-efficacy

According to the self-determination theory, in order to achieve optimal functioning and experience continual personal growth, one must consistently satisfy three essential psychological needs, namely, autonomy, competence, and relatedness (Deci and Ryan, 2008; Fang et al., 2021). Previous studies found a link between employees' perceptions of their supervisors' support and their ability to balance work and family life (Chambel et al., 2022). Subordinate self-efficacy independently mediates the relationship between FSSB and self-rated performance (Straub, 2012). By providing support and creative management methods to balance work and non-work issues, family-supportive supervisors can update and boost female employees' competence, making them have infinite faith in completing tasks and facing troubles at work and home, thereby assisting employees in adapting to challenging work requirements and finding additional opportunities for career development. A woman with strong self-efficacy has a positive mental attitude that will be able to take full advantage of resources and support given by the supervisor to develop her career and deal with problems in work effectively, which could lead to high personal achievement and organizational commitment and engagement, and low turnover intentions. Hence, the relationship described earlier is given in the following hypothesis:

*Hypothesis 4: The relationship between FSSB and career sustainability is mediated by self-efficacy*.

## Methods

For this study, we adopted a mixed-method approach, a more strategic exploration of relevant issues (Creswell, 2014). A qualitative approach primarily explores specific phenomena and covers fundamental constructions for a holistic understanding of the mechanism, while a quantitative study examines the hypotheses and hypothetical relationships (Venkatesh et al., 2013). With this line of thought, in the first phase of the survey, we proceeded with the online and offline interviews with a small sample to acquire a deeper understanding of our research question. Then, a qualitative analysis was conducted to identify the hypothetical model preliminarily based on the result of the interviews. In the second phase of the survey, a questionnaire survey was conducted online with a larger sample to verify the theoretical relationships by a quantitative examination. To a large extent, a mixed-method approach enables the diversification of data collection to produce more robust results.

### Study 1: Qualitative research

#### Sample and data collection

From November to December 2021, online and offline interviews were conducted with five female employees from three different e-commerce companies, which were located in Kunming, Shanghai, and Hangzhou. First was a face-to-face interview with the company in Kunming, which deals in the production and sales of telescopes. Our interaction with the two other companies that deal in new media operations and clothing sales were online. All interview participants were aware of and consented to the presentation of their relevant information in this research, and details of demographics are illustrated in Table 1. In the study, we used “Interviewee+number” instead of interviewees' names due to consideration of the ethical issues.

**Table 1:** Demographic information of interviewees.

**No**	**Interviewee**	**Education**	**Age**	**Tenure (yrs)**	**Marital status**	**Position**	**Location**
1	Interviewee 1	Bachelor	54	14	Married	Accountant	Kunming, China
2	Interviewee 2	College	46	10	Married	Workshop manager	Kunming, China
3	Interviewee 3	Junior college	33	4	Married	Worker	Kunming, China
4	Interviewee 4	Bachelor	29	6	Unmarried	Planning Director	Shanghai, China
5	Interviewee 5	Bachelor	24	1	Unmarried	Sales Manager Assistant	Hangzhou, China

The interview used the following questions:

1. What kind of leadership style do you think your supervisor has?
2. Has your supervisor ever supported you when you are in difficulties at work? What kind of support have you ever received from your supervisor? Does this support make you more confident to complete the task?
3. Have you ever experienced work–family conflict?
4. Does your supervisor care about supporting you with family difficulties?
5. How do you perceive the relationship between your supervisor's supportive behavior and your career sustainability?

Key findings of qualitative research:

First, the outcomes of interviews show that female employees value the care and support offered by their leaders. For instance, interviewee 5, who was unmarried at the time, stated “I appreciate my supervisor for allowing me to arrive late and leave early during the days my mom was in the hospital”. Interviewee 4 said, “my supervisor shows respect to the female employees. At important banquets, he exempts ladies, contrary to the Chinese norms, from taking wine if they are not willing to”.

Furthermore, it reveals from the interview that work and family-supportive behaviors from supervisors positively affect female employee career sustainability. Interviewee 2 told a story about how her supervisor drove her to a distant destination at night for an urgent work issue instead of asking her to take a bus, which confirmed her determination to continue her career in that company. Interviewee 3 stated, “I once decided to resign because of a misunderstanding with my immediate superior on a salary raise issue, fortunately, the top manager communicated intervened, so I stayed here till now”. Interviewee 4 said, “I love this job because my leader allows me to accomplish the project flexibly, I can freely arrange my working time and place.”

Third, according to the interview, female respondents agree that work–family-supportive behaviors improve females' self-efficacy. Interviewee 1 said, “As an older accountant, I am grateful for the support and understanding from my boss over these years, he trusts my working competence and gives me the autonomy to make decisions, which makes me feel confident and get a sense of achievement when tackled with the difficulties.” Interviewee 5 also stated, “I have learned a lot from my supervisor, she always sets challenges for me and shows a good example of how to communicate with consumers. I feel like my capability is constantly improving”.

It can be concluded from the aforementioned qualitative research results that hypothesis 1 and hypothesis 2 proposed in this research are reasonable and consistent with the feedback from interviewees. However, we had not obtained the direct validation of hypothesis 3 and hypothesis 4 from the interviews. Hence, following the small sample qualitative research, the quantitative research was conducted with more samples for further verification of all hypotheses in the next section.

### Study 2: Quantitative research

#### Sample and data collection

From January to April 2022, an online survey of employees was conducted using “wjx.cn” (www.wjx.cn/), which is a well-known professional online survey service in China. The instrument of this research was posted on this website. The objects of the survey were female employees who work in the e-business industries in China. In total, 281 valid questionnaires were obtained, with 21 excluded due to lack of completeness and normative requirements (Su et al., 2021). A valid response rate is 93%. According to verified sample statistics, 23% of the participants were younger than 25 years, 66% were between 26 and 40 years old, and 11% were older than 41 years. There were 73% of married women and 27% unmarried. Furthermore, 18% had master's degrees, 67% had bachelor's degrees, and 15% got an associated degree or below. To reduce the common method variance problem caused by the continuous use of the same rating scale and give respondents sufficient time to move between pages, the questionnaire was paginated (Podsakoff et al., 2003).

### Measures

In this study, FSSB is considered an independent variable, and career sustainability is the dependent variable. Moreover, self-efficacy is used as a mediating variable. The FSSB scale developed by Hammer et al. (2013) contains four items measured by the six-point Likert scale (1 = strongly disagree to 6 = strongly agree). They include “Your supervisor makes you feel comfortable talking to him/her about your conflicts between work and non-work,” “Your supervisor works effectively with employees to creatively solve conflicts between work and non-work,” and “Your supervisor organizes the work in your department or unit to jointly benefit employees and the company.” The dimension of “role modeling” in FSSB was deleted in this study because in the interviews, we found that male supervisors do not set a significant example for female employees when it comes to balancing work and non-work (family life) due to the gender difference. The number of male supervisors in the sampled e-commerce firms was more than the number of female supervisors. The outcomes of this study point out that the alpha value of FSSB is 0.9, which is up to an acceptable standard. Therefore, the items of FSSB were reliable and valid.

The scale by Chin et al. (2022) for career sustainability is fairly recent, yet it is consistent with the design of our study. Items of career sustainability scale had been adopted and modified using the six-point Likert scale (1 = strongly disagree to 6 = strongly agree). The sample items contain “My career allows me to continuously learn new things,” “My career gives me a lot of flexibility,” and “My career makes me feel happy because I use my resources well”. The outcome of this research shows that the alpha value of career sustainability is 0.93, which is up to an acceptable standard. Thus, the items of career sustainability were reliable and valid.

The mediating variable, the self-efficacy scale (SE), was developed by Schwarzer et al. (1997). The scale has been commonly utilized in the field of management and cognitive psychology. All items of self-efficacy were also measured using the six-point Likert scale (1 = strongly disagree to 6 = strongly agree). Sample items include “l can always manage to solve difficult problems if I try hard enough,” “It is easy for me to stick to my aims and accomplish my goals,” and “I am confident that I could deal efficiently with unexpected events”. The outcomes of this research show that the alpha value of self-efficacy is 0.92, which is up to an acceptable standard. Therefore, the items of self-efficacy were reliable and valid.

### Procedures

The survey was carried out in Chinese to suit the Chinese respondents. The data were collected with the hypotheses as the foundation, and participants willingly agreed to respond anonymously. We explained the survey process's confidentiality when undertaking the formal online survey. There were no confidential information and sensitive topics involved in any of the questions.

## Results

### Measurement model

This work used SmartPLS 3.0 for the direct and indirect correlation hypothesis analysis. We measured and assessed the structural model using SEM-PLS as it is extensively used in marketing management, information management, organizational management, human resource management, and tourist management research (Yang and Lin, 2014; Suen et al., 2019). It is a practical tool for revealing intricate interactions between observable and latent variables. Cronbach's alpha and composite reliability (CR) were employed to evaluate the reliability. As seen in Table 2, Cronbach's alpha values for each dimension varied from 0.90 to 0.93 (FSSB and CS, respectively), and CR were all 0.94 (FSSB, CS, SE). In addition, rho A, a new indicator coefficient introduced in Smart PLS3.0, was evaluated to correct the estimation of the measured structure, which ranged from 0.90 to 0.93 (FSSB and CS, respectively) and was significantly over the required threshold of 0.7. These findings revealed that the internal consistency of the measurement in this study was acceptable (Hair et al., 2017).

**Table 2:** Analysis results of factor loading, Cronbach's alpha, composite reliability, and AVE.

**Construct**	**Items**	**Factor loading**	**α**	**Rho-A**	**CR**	**AVE**
Family-supportive supervisor behavior (FSSB)	FSSB1	0.91^***^	0.90	0.90	0.94	0.84
	FSSB2	0.91^***^				
	FSSB3	0.92^***^				
Career sustainability (CS)	CS1	0.78^***^	0.93	0.93	0.94	0.58
	CS2	0.78^***^				
	CS3	0.79^***^				
	CS4	0.76^***^				
	CS5	0.72^***^				
	CS6	0.78^***^				
	CS7	0.75^***^				
	CS8	0.73^***^				
	CS9	0.77^***^				
	CS10	0.76^***^				
	CS11	0.75^***^				
	CS12	0.74^***^				
Self-efficacy (SE)	SE1	0.92^***^	0.92	0.92	0.94	0.81
	SE2	0.89^***^				
	SE3	0.88^***^				
	SE4	0.90^***^				

*** means T > 1.96.

For the convergent validity, factor loadings of all items were more than 0.7, which shows significance. The average variance extracted (AVE) values ranges from 0.58 to 0.84 (>5.0) (CS and FSSB, respectively). As a result, the convergent validity of these measures was found to be satisfactory. The variance inflation factor (VIF) values range from 1.0 to 1.49, which were below 5, and thus, the results meet the requirements (Hair et al., 2017). The discriminant validity of constructs was assessed using the method proposed by Fornell and Larcker (1981). As presented in Table 3, the square roots of the AVE values were all larger than the correlation coefficients, manifesting that discriminant validity of the measures is satisfactory (Fornell and Larcker, 1981).

**Table 3:** Analysis of discriminant validity (Fornell-Larcker Criterion).

**Construct**	**Supportive leadership**	**Career Sustainability**	**Self-efficacy**
FSSB	0.91		
CS	0.74	0.76	
SE	0.57	0.68	0.90

### Structural model

**Figure 1:**
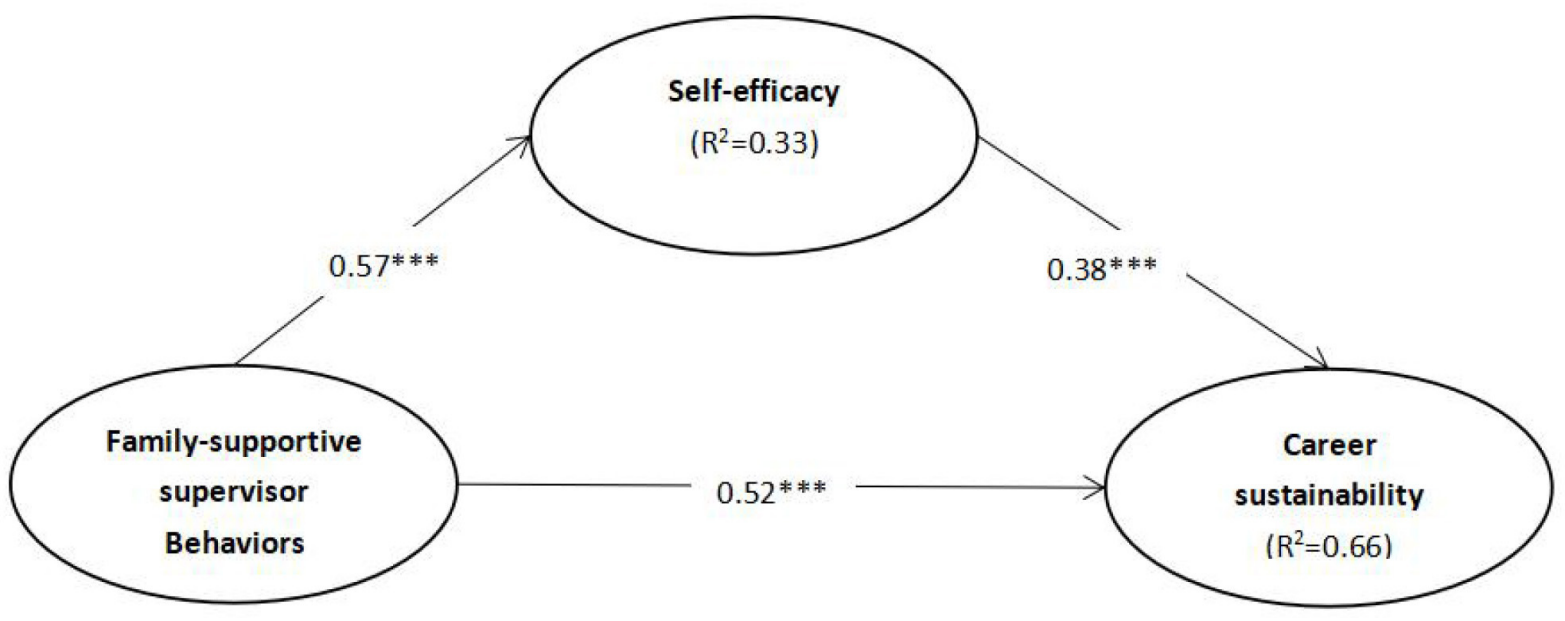
PLS results outcomes. ***T > 1.96.

To test the hypotheses and evaluate the PLS results, the bootstrap resampling method in SmartPLS was used, and the responses were resampled 5,000 times (Hair et al., 2017). In Table 4, the overall *R*^2^ value was 0.65, manifesting that the research model adequately explains 65% of the variance in career sustainability. The results support hypotheses H1, H2, H3, and H4. The results suggest that family-supportive supervisor behaviors had a positive effect on female employee career sustainability (H1, β = 0.52, *P* < 0.01), family-supportive supervisor behaviors positively related to self-efficacy (H2, β = 0.57, *P* < 0.01), and self-efficacy significantly affected career sustainability (H3, β = 0.38, *P* < 0.01). At last, the mediating role of self-efficacy between family-supportive supervisor behaviors and career sustainability was verified (H4, β = 0.22, *P* < 0.01). The PLS results of the research model are presented in Figure 1.

**Table 4:** Serial mediation results.

**Hypothesis**	**Effect**	***T*** **-value**	***P*** **-value**	**Result**
H1: FSSB → CS	0.52	11.18	0.00	Significant
H2: FSSB → SE	0.57	11.21	0.00	Significant
H3: SE → CS	0.38	7.6	0.00	Significant
H4: FSSB → SE → CS	0.22	6.41	0.00	Significant

CS, career sustainability; SE, self-efficacy.

## Discussion

### Implication for theories

Based on the application of the mixed-method approach, this study verified the significance of the positive correlations of FSSB-CS, FSSB-SE, and SE-CS. A number of prior research studies focused on the relationship between leadership, individual performance, and organizational performance, but little attention had been paid to the direct link between FSSB and individuals' career sustainability. Meanwhile, most relevant research studies used a single method to generalize the results. This study filled the gap and made some innovations that validated the direct and indirect relationships between FSSB and career sustainability by using the mixed-method approach and focusing on the female groups in the context of Chinese culture.

Also, this study enriches the existing literature on career sustainability by addressing self-efficacy as a mediator between FSSB and career sustainability. Previous studies mainly explored the influence of FSSB on personal performance based on the social exchange theory (Fan et al., 2018; Shi et al., 2019) but ignored the individuals' deeper motivation. In this study, we explored the deeper motivation of female workers in study 1, and self-efficacy is selected as the mediating variable, revealing that the impact of FSSB on female career can be transmitted through self-efficacy. The results report a novel perspective on the research of relations between FSSB and a person's career choice.

### Practical implication

Some useful practical implications for e-commerce organizations are extracted from study 1 and study 2. From the positive impact of FSSB in the workplace, we posit that FSSB is encouraged, especially in female employee-dominated companies (Yu et al., 2022). For example, work location, time flexibility, and effective resource provision tremendously contribute to female workers' work–family balance.

Furthermore, healthy psychological and physiological assistance are vital for career sustainability (Fang et al., 2021). Unfortunately, most leaders focus more on performance than the mental state, but women are more emotional. Since a positive psychological state is crucial to making a career more sustainable (Bouleh et al., 2022), supervisors should have an insight into the female employees' psychological state to help them keep a positive attitude toward their job and family life.

Moreover, to improve female self-efficacy, managers can take effective measures such as giving female staff more opportunities to challenge themselves, through success or failure experiences, to upgrade their job skills. They can also provide chances for employees to learn from each other to accumulate more experience and skills. Female employees could also be encouraged to use achievements made in order to set up their confidence and strengthen their sense of self-efficacy, resulting in their desire to persist in a long-term career.

## Limitations and future research

Despite the useful contribution to the management literature, this research counts some limitations. First, the sample size was relatively small, and married women predominated in the sample. A larger sample size with a high proportion of unmarried women could be used for evaluating the proposed model in future studies. Second, the samples were mainly chosen from e-commerce enterprises with a Chinese cultural background. Future studies can investigate the situation from a broader cross-cultural and cross-generational perspective. Third, this study took self-efficacy as a mediator in the relationship between FSSB and career sustainability. The explorations of other mediators at the individual level and organizational levels could be involved in future research. In conclusion, our findings hint that future studies may give more attention to training supervisors to cultivate and form FSSB and, in turn, to achieve a win–win situation between the organization and employees.

## Data availability statement

The original contributions presented in the study are included in the article/supplementary material, further inquiries can be directed to the corresponding author/s.

## Ethics statement

Ethical review and approval were not required for the study on human participants in accordance with the local legislation and institutional requirements. The participants provided consent to participate in this study.

## Author contributions

HL conceived and designed the research, provided guidance throughout the entire research process, and wrote the hypotheses development. HL and GA wrote the main part of the manuscript. FL and TC collected the data and wrote the Methods section. MT and GA offered modification suggestions. FL and TC conducted the online survey and helped analyze the data. All authors contributed to the article and approved the submitted version.

## Conflict of interest

The authors declare that the research was conducted in the absence of any commercial or financial relationships that could be construed as a potential conflict of interest.

## Publisher's note

All claims expressed in this article are solely those of the authors and do not necessarily represent those of their affiliated organizations, or those of the publisher, the editors and the reviewers. Any product that may be evaluated in this article, or claim that may be made by its manufacturer, is not guaranteed or endorsed by the publisher.
